# ﻿Molecular and morphological data reveal two new polypores (Polyporales, Basidiomycota) with reddish brown to orange basidiomata from China

**DOI:** 10.3897/mycokeys.107.126176

**Published:** 2024-07-15

**Authors:** Xin Zhang, Hong-Min Zhou, Masoomeh Ghobad-Nejhad, Hong-Gao Liu, Josef Vlasák, Yu-Cheng Dai, Yuan Yuan

**Affiliations:** 1 State Key Laboratory of Efficient Production of Forest Resources, School of Ecology and Nature Conservation, Beijing Forestry University, Beijing 100083, China Beijing Forestry University Beijing China; 2 College of Biodiversity Conservation, Southwest Forestry University, Kunming 650224, China Southwest Forestry University Kunming China; 3 Department of Biotechnology, Iranian Research Organization for Science and Technology (IROST), Tehran 3353-5111, Iran Iranian Research Organization for Science and Technology (IROST) Tehran Iran; 4 Yunnan Key Laboratory of Gastrodia and Fungi Symbiotic Biology, Zhaotong University, Zhaotong 657000, China Zhaotong University Zhaotong China; 5 Biology Centre of the Academy of Sciences of the Czech Republic, Branišovská 31, CZ-370 05 České Budějovice, Czech Republic Biology Centre of the Czech Academy of Sciences České Budějovice Czech Republic

**Keywords:** Phlebioid clade, phylogeny, taxonomy, wood-rotting fungi

## Abstract

Two taxonomically controversial polypore genera with reddish brown to orange basidiomata that stain reddish with KOH solution, *Aurantiporus* and *Hapalopilus*, are revised based on additional sampling, morphological examination, and phylogenetic analysis of a combined dataset of ITS1-5.8S-ITS2-nLSU sequences. *Hapalopilus* is a monophyletic genus belonging to Phanerochaetaceae, whereas *Aurantiporus* is a polyphyletic genus belonging to Meruliaceae. *Hapalopilus* and *Aurantiporus* s. str. are circumscribed, and two new species – *Aurantiporusorientalis* and *Hapalopilustabuliformis* – are described and illustrated from temperate China. In addition, four new combinations, viz. *Aurantiporusalboaurantius*, *A.mutans*, *A.tropicus* and *Luteoporiaalbocitrina*, are proposed based on morphology and phylogenetic analysis. The relationships between *Aurantiporus* and *Hapalopilus* are discussed.

## ﻿Introduction

Polypores are important wood-decaying fungi and have distribution in all the forest ecosystems; around 2670 polypores have been reported worldwide and some have economic values (Papp and Dai 2022; Wu et al. 2019, 2022; Yuan et al. 2023; Zhao et al. 2024). *Aurantiporus* Murrill and *Hapalopilus* P. Karst. are polypore genera with limited species producing reddish brown to orange basidiomata and belonging to the phlebioid clade of Polyporales (Chen et al. 2021). *Aurantiporus* was treated as a synonym of *Hapalopilus* by Ryvarden (1991), and its type species was accepted as *Hapalopiluscroceus* (Pers.) Donk (Donk 1933). Until now, the name of *Hapalopiluscroceus* is still accepted by some mycologists (Langer et al. 2015; Ryvarden and Melo 2017; Redr et al. 2020).

*Aurantiporus* was typified as *Polyporuspilotae* Schwein. (Murrill 1905) and is characterized by annual, bright-colored basidiomata staining more or less red with KOH solution, a monomitic hyphal system with agglutinated clamped hyphae, and hyaline, smooth, ellipsoid basidiospores which are negative in Cotton Blue (Niemelä et al. 2012; Rivoire 2020). Jahn (1974) pointed out that *Hapalopiluscroceus* with different pigmentation and denser basidiomata consistency should be addressed in *Aurantiporus*. Moreover, recent phylogenetic studies showed that *H.croceus* was nested in the Meruliaceae clade and was distantly related to the type of *Hapalopilus* belonging to Phanerochaetaceae (Dvořák et al. 2014; Koszka and Papp 2020).

The genus *Hapalopilus* was established by Karsten (1881) and typified by *Polyporusnidulans* Fr. (= *Hapalopilusrutilans* (Pers.) Murrill 1904). Morphologically, the genus is known by its distinctive annual, colorful, soft basidiomata with a reddish to violet coloration upon contact with KOH solution, a monomitic hyphal system, generative hyphae bearing clamp connections, and hyaline, thin-walled, smooth basidiospores which are negative in Melzer’s reagent and Cotton Blue (Miettinen et al. 2016; Ryvarden and Melo 2017; Chen et al. 2021). The color change in KOH solution has been attributed to the presence of polyporic acid in *H.rutilans*, which renders the fungus poisonous (Kraft et al. 1998; Villa et al. 2013).

Previously, most polypore species with reddish KOH reaction were placed in *Hapalopilus* on a morphological basis (Gilbertson and Ryvarden 1986; Zmitrovich et al. 2006; Ryvarden and Melo 2017) and therefore *Hapalopilus* was considered polyphyletic in early molecular studies (Ko et al. 2001). Niemelä et al. (2005) established *Erastia* Niemelä & Kinnunen to accommodate *H.salmonicolor* (Berk. & M.A. Curtis) Pouzar, and recently two additional species, *H.aurantiacus* (Rostk.) Bondartsev & Singer and *H.ochraceolateritius* (Bondartsev) Bondartsev & Singer, were combined into this genus (Zmitrovich 2018; Zíbarová et al. 2021). Furthermore, the phylogenetic studies demonstrated that *H.ochraceolateritius* belonged to Irpicaceae (Justo et al. 2017; Chen et al. 2021; Li et al. 2022). However, the phylogenetic position of *Erastia* and its type species was uncertain. Based on molecular and morphological analyses, Miettinen et al. (2016) redefined *Hapalopilus* as monophyletic with a narrow concept that included its type species and three additional species within Phanerochaetaceae. Although this revised classification enhanced the clarity of phylogenetic relationships within *Hapalopilus* in Phanerochaetaceae, other species initially described in *Hapalopilus* were found in different clades of Polyporales, highlighting unresolved taxonomic issues (Ko et al. 2001; Justo et al. 2017; Chen et al. 2021).

To better understand the morphological variation and phylogeny of the above bright-colored polypores with basidiomata staining red in KOH solution, and especially the uncertain phylogenetic position of some species in *Aurantiporus* and *Hapalopilus*, we examined specimens from Asia and North America. Based on morphology and new molecular data, we provide an updated phylogeny of *Aurantiporus* and *Hapalopilus*. As a result, two new species are described and four new combinations are proposed in this study.

## ﻿Materials and methods

### ﻿Morphological studies

The specimens used in this study are deposited at the Fungarium of the State Key Laboratory of Efficient Production of Forest Resources, Beijing Forestry University, China (BJFC), the private herbarium of Josef Vlasák (JV), and the National Museum Prague of Czech Republic (PRM). Macro-morphological descriptions are based on field notes and voucher specimens. Our morphological studies follow Miettinen et al. (2016) and Westphalen et al. (2022). The following abbreviations are used: IKI = Melzer’s reagent, IKI−= neither amyloid nor dextrinoid, CB = Cotton Blue, CB−= acyanophilous in Cotton Blue, L = arithmetic average length of all measured spores, W = arithmetic average of all measured spore width, Q = L/W ratios among the studied specimens, and n (a/b) = number of spores (a) measured from a given number of specimens (b). Color terms follow Anonymous (1969) and Petersen (1996).

### ﻿Molecular studies and phylogenetic analysis

Total genomic DNA was extracted from dried specimens using the CTAB plant genomic DNA extraction kit DN14 (Aidlab Biotechnologies Co., Ltd, Beijing, China), following the manufacturer’s guidelines with some modifications (Shen et al. 2019; Sun et al. 2020). The ITS1-5.8S-ITS2 region was amplified using the primer pairs ITS4 and ITS5, and the nLSU region was amplified using the primer pairs LROR and LR7 (White et al. 1990). The PCR procedures for the ITS1-5.8S-ITS2 and nLSU regions followed Wang et al. (2023) and Zhang et al. (2023). The PCR products were purified and sequenced at the Beijing Genomics Institute, China (BGI) with the same primers. All newly generated sequences were submitted to GenBank and are listed in Table 1.

**Table 1.** Taxa information and GenBank accession numbers of sequences used in this study.

Species name	Samples/Voucher	Country	GenBank Accession no.	
			ITS no.	nLSU no.
*Alboefibulabambusicola*	Chen 2304 (holotype)	China	MZ636926	MZ637091
*Aurantiopileusmayaensis*	MCW 373/12	Brazil	OL630487	OL630487
*A.mayaensis*	TJB10228 (holotype)	Belize	HM772140	HM772139
*A.mayaensis*	JV1504/128	Costa Rica	KT156706	/
*Aurantiporusalbidus*	CIEFAP-117	Argentina	KY948739	KY948848
*A.albidus*	F32	Argentina	MT076170	/
*A.* ‘*albidus*’	Cui 16664	Australia	ON682353	ON680805
*A.* ‘*albidus*’	Cui 16665	Australia	ON682354	ON680806
*A.alboaurantius*	Cui 2877	China	KF845954	KF845947
*A.alboaurantius*	Cui 4136 (holotype)	China	KF845955	KF845948
*A.croceus*	H6-27	Lithuania	MH571407	/
*A.croceus*	VPapp 300518-1	Hungary	MT876120	/
*A.croceus*	BRNM737561	Czech	JQ821320	JQ821317
*A. ‘croceus*’	TVR 7	USA	MW020539	/
*A. ‘croceus*’	PUL00031376	USA	OM747650	/
*A. ‘croceus*’	57362583	USA	OM473901	/
*A.mutans*	JV0509/123	USA	MN318460	/
*A.mutans*	JV0309/83a	USA	MN318458	/
*A.mutans*	JV0309/83b	USA	MN318459	/
***A.orientalis***	**Dai 23714 (holotype)**	**China**	**PP702380**	**PP623071**
*A.pseudoplacentus*	Miettinen 18997	USA	KY948744	KY948902
*A.pseudoplacentus*	PRM 899297 (holotype)	USA	JN592496	JN592504
*A.pulcherrimus*	MR80	Argentina	OL630488	OL630488
*A.roseus*	Dai 13573 (holotype)	China	KJ698635	KJ698639
*A.roseus*	CLZhao 4762	China	PP392925	/
*A.* sp. (*A.* ‘*croceus*’)	Miettinen 16483	Malaysia	KY948745	KY948901
***A.* sp. (*A.* ‘*priscus*’)**	**Dai 4686**	**China**	**PP916606**	/
*A.* sp. (*A.* ‘*priscus*’)	Dai 22793	China	ON413717	ON413719
*A.* sp. (*A.* ‘*priscus*’)	VS6295	Russia	MN318461	/
*A.tropicus*	JV1707/5T	Costa Rica	MN318455	/
*A.venustus*	MCW 391/12	Brazil	OL630489	OL635577
*Bjerkanderaadusta*	HHB-12826-Sp	USA	KP134983	KP135198
*Byssomeruliuscorium*	FP-102382	USA	KP135007	KP135230
*Ceriporiagossypinum*	Dai 23392 (holotype)	China	OQ476824	OQ476770
*C.viridans*	Dai 17003	China	OQ476847	OQ476790
*Ceriporiopsisgilvescens*	BRNM 710166	Czech	FJ496684	FJ496720
*C.semisupina*	Cui 10222 (holotype)	China	KF845956	KF845949
*C.semisupina*	Cui 7971	China	KF845957	KF845950
*Crustodontiachrysocreas*	HHB-6333-Sp	USA	KP135358	KP135263
*Crystallicutisserpens*	HHB-15692-Sp	USA	KP135031	KP135200
*Efibulatropica*	He 6008	China	MW580947	MW580937
*Erastiaaurantiaca*	BR4112	France	MN318464	/
***E.aurantiaca***	**Dai 18399**	**Vietnam**	**PP715440**	/
*E.aurantiaca*	Gustafson176	Unknown	AY986499	/
***E.ochraceolateritia***	**Dai 23109**	**China**	**PP715441**	/
*E.ochraceolateritia*	JV1609/12TDK	Czech	MN318463	/
*E.ochraceolateritia*	Miettinen 16992	USA	KY948741	KY948891
*E.ochraceolateritia*	VS4749	Russia	MN318462	/
*E.salmonicolor*	FLAS-F-61674	USA	MH212041	/
*E.salmonicolor*	JV0904/46	USA	JN592500	JN592507
*E.salmonicolor*	MC13	USA	MW619631	/
*Gloeoporushainanensis*	Dai 15268 (holotype)	China	KU360401	KU360411
*G.thelephoroides*	JV 1808 26	French Guiana	OQ476858	OQ476799
*Hapalopiluseupatorii*	F. Dammrich 10744	Germany	KX752620	KX752620
*H.eupatorii*	K 132752	UK	KX008364	KX081076
*H.percoctus*	H 7008581 (holotype)	Botswana	KX752597	KX752597
*H.ribicola*	H 6045691	Finland	KX752616	/
*H.ribicola*	H 6045697	Finland	KX752617	/
*H.rutilans*	Dai 23591	China	OL469801	OL469800
*H.rutilans*	H 6012735	Finland	KX752614	/
*H.rutilans*	H 6013411	Finland	KX752615	/
***H.tabuliformis***	**Dai 24535**	**China**	**PP715438**	**PP623072**
***H.tabuliformis***	**Dai 24540 (holotype)**	**China**	**PP715439**	**PP623073**
*Heterobasidionannosum*	Dai 20962	Belarus	ON417163	ON417213
*Irpexlacteus*	Dai 11230	China	OQ476863	OQ476805
*Leptoporusmollis*	Dai 21062	Belarus	MW377302	MW377381
*Luteoporiaalbocitrina*	JV1704/103	Costa Rica	MN318457	/
*L.albocitrina*	Dai 19507 (holotype of *L.citriniporia*)	Sri Lanka	MT872218	MT872216
*L.albocitrina*	Dai 19622	Sri Lanka	MT872219	MT872217
*L.albomarginata*	Dai 15229 (holotype)	China	KU598873	KU598878
*L.albomarginata*	Dai 15240	China	KU598874	KU598879
*L.albomarginata*	GC 1702-1	China	LC379003	LC379155
*L.lutea*	CHWC 1506-68	China	MZ636997	MZ637157
*L.lutea*	GC 1409-1	China	MZ636998	MZ637158
*L.straminea*	CLZhao 5794	China	OM897115	OM897114
*L.straminea*	CLZhao 18947 (holotype)	China	MW732407	MW724799
*L.tenuissima*	Dai 20429	China	PP356578	PP356576
*L.tenuissima*	Dai 25825 (holotype)	China	PP356579	PP356577
*Meruliopsistaxicola*	Dai 22625	China	OL457966	OL457436
*Mycoaciafuscoatra*	HHB-10782-Sp	USA	KP135365	KP135265
*M.nothofagi*	HHB-4273-Sp	USA	KP135369	KP135266
*Odoriaalborubescens*	BP106943	Hungary	MG097864	MG097867
*O.alborubescens*	BRNU 627479	Czech	JQ821319	JQ821318
*O.alborubescens*	PC 0706595	France	MG097863	/
*Pappiafissilis*	BRNM 699803	Czech	HQ728292	HQ729002
*P.fissilis*	MUcc 814	Czech	HQ728291	HQ729001
*P.fissilis*	HHB-9530-Sp	USA	KY948774	/
*Phaeophlebiopsiscaribbeana*	HHB-6990	USA	KP135415	KP135243
*Phanerochaetechrysosporium*	HHB-6251-Sp (holotype)	USA	KP135094	KP135246
*P.inflata*	Cui 7712	China	JX623930	JX644063
*Phanerochaetellaangustocystidiata*	Wu 9606-39	China	MZ637020	GQ470638
*Phlebiaaustroasiana*	Dai 17556 (holotype)	China	ON135439	ON135443
*P.poroides*	CLZhao 16121 (holotype)	China	MW732405	MW724797
*P.radiata*	AFTOL-484	Unknown	AY854087	AF287885
*P.rufa*	FBCC297	Sweden	LN611092	LN611092
*P.setulosa*	HHB-6891-Sp	USA	KP135382	KP135267
*P.tomentopileata*	CLZhao 9563 (holotype)	China	MT020765	MT020743
*P.tremellosa*	FBCC82	Finland	LN611124	LN611124
*Phlebicoloratabrevispora*	FBCC1463 (holotype)	USA	LN611135	LN611135
*Phlebiopsisgigantea*	FCUG 1417	Norway	MZ637051	AF141634
*Resiniporuspseudogilvescens*	Wu 1209-46	China	KY688203	MZ637268
*Rhizochaetefouquieriae*	KKN-121	USA	AY219390	GU187608
*Skeletocutisamorpha*	Miettinen 11038	Finland	FN907913	FN907913
*S.chrysella*	Miettinen 9472	Finland	FN907916	FN907916
*Stereumhirsutum*	FP-133888	Unknown	AY854063	/
*Trametopsiscervina*	TJV-93-216T	USA	JN165020	JN164796
*Tyromyceschioneus*	Miettinen 7487	Finland	HQ659244	HQ659244

Newly generated sequences for this study are in bold.

**Figure 1.**
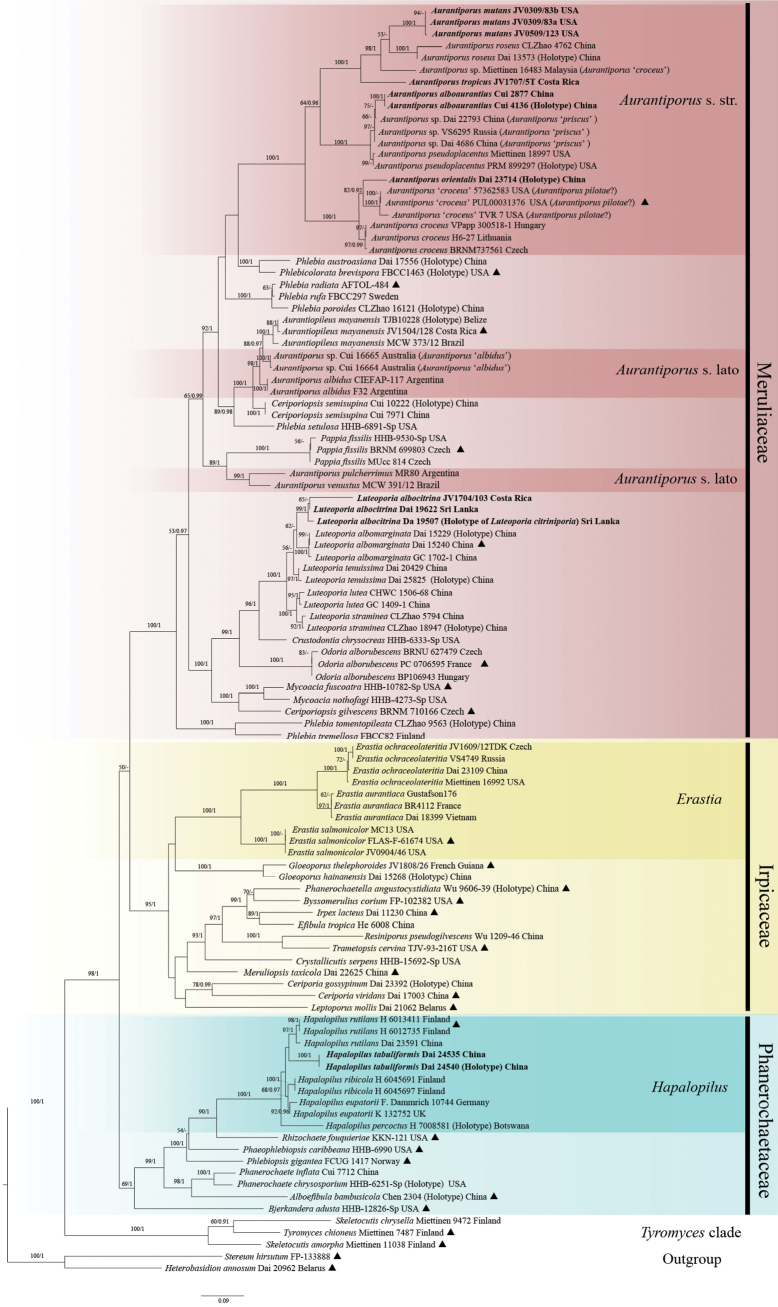
Maximum likelihood tree (ML) illustrating the phylogeny of the phlebioid clade within the Polyporales based on a combined ITS1-5.8S-ITS2-nLSU dataset. Branches are labelled with ML bootstrap values higher than 50% and Bayesian Posterior Probabilities (BPP) more than 0.90. New species and new combinations are in bold. Black triangles represent the generic types.

Sequences generated for this study and additional sequences downloaded from GenBank were partitioned to ITS1, 5.8S, ITS2 and nrLSU, and then aligned separately using MAFFT v.74 (http://mafft.cbrc.jp/alignment/server/; Katoh et al. 2017) with the G-INS-I iterative refinement algorithm. Following manual optimization in BioEdit 7.0.5.3 (Hall 1999), the separate alignments were concatenated using PhyloSuite v. 1.2.3 (Zhang et al. 2020; Xiang et al. 2023). The combined ITS1-5.8S-ITS2-nLSU dataset was analyzed to confirm the phylogenetic position of target species within the phlebioid clade of Polyporales (Fig. 1). Sequences of *Heterobasidionannosum* (Fr.) Bref. and *Stereumhirsutum* (Willd.) Pers. were used as outgroups following Chen et al. (2021) and Liu et al. (2023). The resulting alignment was deposited at TreeBase (submission ID 31520; Reviewer access URL: http://purl.org/phylo/treebase/phylows/study/TB2:S31520?x-access-code=49bae894df9bffc4280d3da656868775&format=html). Maximum Likelihood (ML) and Bayesian Inference (BI) methods were used for the phylogenetic analysis. ModelFinder v. 2.2.0 with Corrected Akaike information criterion (AICc) was applied to estimate the best-fit partition scheme and evolutionary model for BI (Kalyaanamoorthy et al. 2017).

Maximum Likelihood (ML) analysis was performed in RAxML v.8.2.10 (Stamatakis 2014). All parameters in the ML analysis used default settings, and statistical support values were obtained using rapid bootstrapping with 1000 replicates.

Bayesian Inference (BI) analysis was run with four chains for two runs and performed for two million generations sampling every 1000 generations in MrBayes v3.2.7 (Ronquist et al. 2012), until the split deviation frequency value was less than 0.01. A burn-in of 25% was used before computing the consensus tree.

Trees were viewed in FigTree v. 1.4.4 (http://tree.bio.ed.ac.uk/software/figtree/). Branches that received bootstrap support for ML and Bayesian Posterior Probabilities (BPP) greater than or equal to 75% (ML) and 0.95 (BPP) were considered to be significantly supported.

## ﻿Results

### ﻿Molecular phylogeny

The combined ITS1-5.8S-ITS2-nLSU dataset of the phlebioid clade included sequences from 107 specimens (Phanerochaetaceae, Irpicaceae, and Meruliaceae) representing 68 taxa and the outgroup (Table 1). ModelFinder suggested SYM+I as the best-fit model for 5.8S, and GTR+F+I+G4 as the best-fit models for ITS1, ITS2, and nrLSU for the Bayesian analysis. BI analysis yielded an almost identical topology to the ML analysis, with an average standard deviation of split frequencies of 0.006514, and thus only the ML tree (Fig. 1) is presented with branch support values for ML and BI when these were greater than or equal to 50% and 0.90, respectively.

The phylogeny of the phlebioid clade (Fig. 1) revealed that *Hapalopilus* is fully supported and clustered within Phanerochaetaceae as a monophyletic genus that includes five species (100% ML, 1.00 BPP; Fig. 1). Similarly, *Erastia* includes three species and forms a monophyletic clade within Irpicaceae (100% ML, 1.00 BPP; Fig. 1). It is also obvious from the genetic analysis that *Aurantiporus* is highly polyphyletic in its current concept, representing three separate clades within Meruliaceae. Notably, the sampled sequences of the so-called *A.croceus* (Pers.) Murrill appear to represent three different species from different continents (USA, Europe, and Malaysia), but all well nested within *Aurantiporus* s. str. (Fig. 1). In addition, two specimens from Northern China, annotated as *Hapalopilustabuliformis*, form a distinct lineage with robust support and are stably nested within *Hapalopilus* (100% ML, 1.00 BPP; Fig. 1). Another specimen from Northeast China, annotated as *Aurantiporusorientalis*, grouped together with so-called *A.croceus* from North America and Europe (the type species of *Aurantiporus*) with robust support (100% ML, 1.00 BPP; Fig. 1). *Hapalopilusalbocitrinus* (Petch) Ryvarden is nested in *Luteoporia* F. Wu et al. and is transferred to the latter genus in the present study. *Ceriporiopsisalboaurantia* C.L. Zhao et al., *Hapalopilusmutans* (Peck) Gilb. & Ryvarden and *H.tropicus* I. Lindblad & Ryvarden nested in the *Aurantiporus* s. str. clade, are transferred to *Aurantiporus* in this study.

### ﻿Taxonomy

#### ﻿ Aurantiporus orientalis

Taxon classificationFungiPolyporalesMeruliaceae

Y.C. Dai, Xin Zhang, Ghobad-Nejhad & Yuan Yuan sp. nov.

9E193D43-F97E-5E29-8EFB-B1142ABBAD70

853592

Figs 2 , 3

##### Holotype.

China, Jilin Province, Antu County, Changbaishan Nature Reserve, on living tree of *Quercusmongolica*, 4 July 2022, Dai 23714 (BJFC 038959).

**Figure 2.**
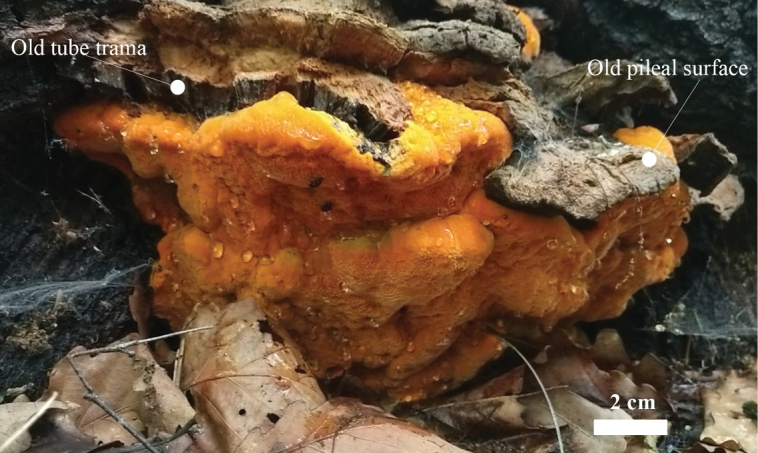
Basidiomata of *Aurantiporusorientalis* (Dai 23714).

##### Etymology.

*Orientalis* (Lat.): refers to the species occurring in East Asia.

**Figure 3.**
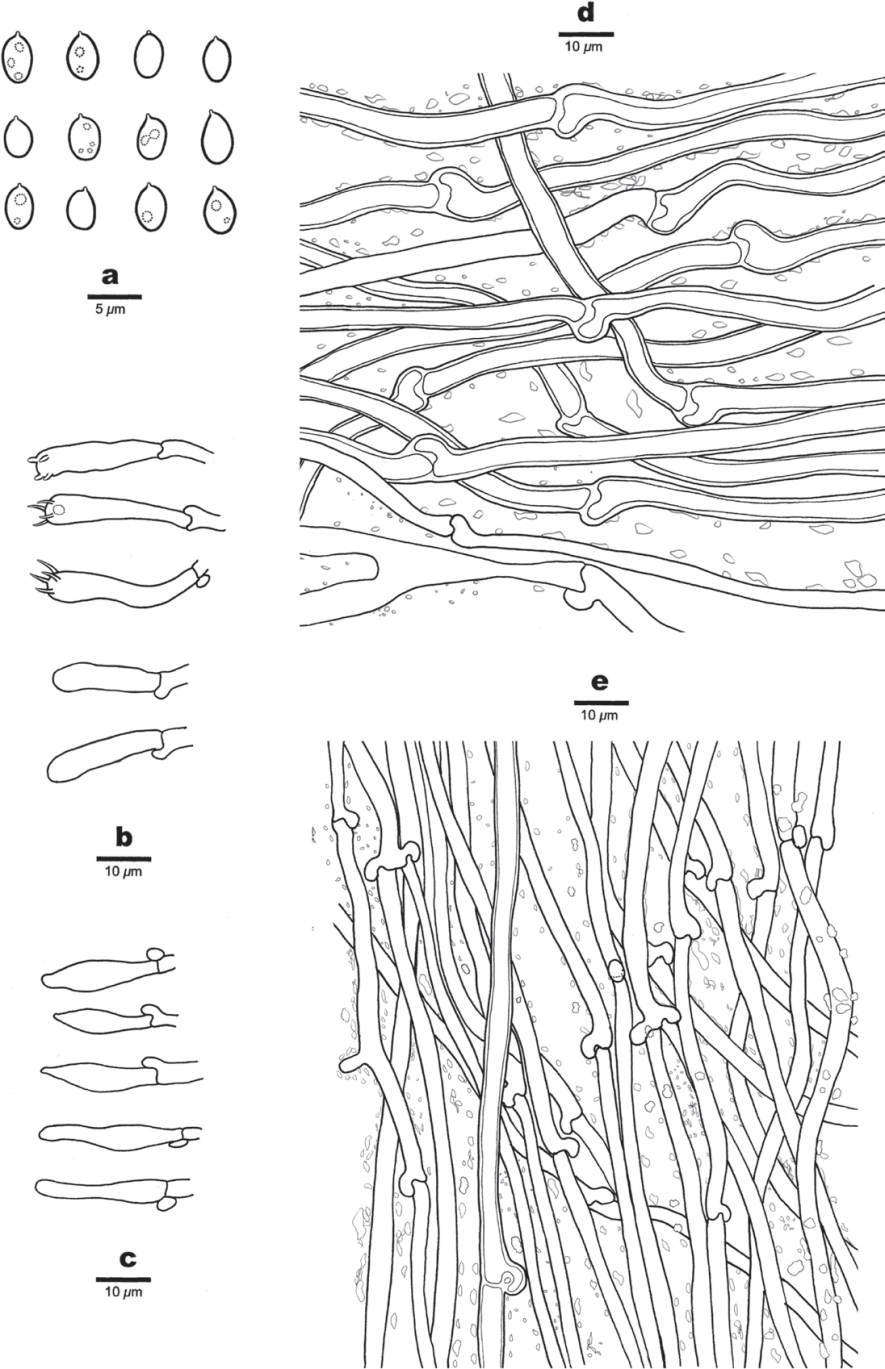
Microscopic structures of *Aurantiporusorientalis* (Dai 23714, holotype) **a** basidiospores **b** basidia and basidioles **c** cystidioles **d** hyphae from context **e** hyphae from tube trama.

##### Diagnosis.

*Aurantiporusorientalis* is characterized by pileate, imbricate, triquetrous basidiomata with apricot-orange pores when fresh, that become honey yellow upon drying and reddish in KOH solution, large pores 1–2 per mm, the presence of cystidioles, broadly ellipsoid basidiospores measuring 3.4–4 × 2.5–3 μm, and growing on *Quercus* in Northeast China.

##### Fruitbody.

Basidiomata annual, pileate, imbricate, inseparable from the substrate, watery to soft corky and without odor or taste when fresh, shrinking and becoming brittle to hard corky upon drying. Pilei triquetrous, projecting up to 10 cm, 15 cm wide and 3 cm thick at base. Pileal surface orange-yellow when fresh, becoming honey-yellow upon drying, matted. Pore surface apricot-orange when fresh, becoming fuscous to date brown upon drying; sterile margin distinct, concolorous with pileal surface, up to 3 mm wide; pores angular to irregular, 1–2 per mm; dissepiments thin, lacerate. Context clay-buff and hard corky when dry, up to 2.5 cm thick, becoming reddish in KOH solution. Tube layer concolorous with pore surface, brittle to rigid, up to 5 mm deep.

##### Hyphal structure.

Hyphal system monomitic; generative hyphae bearing clamp connections, richly encrusted with fine yellowish crystals, IKI–, CB–; tissue becoming reddish in KOH solution.

##### Context.

Generative hyphae hyaline, slightly thick- to thick-walled, occasionally branched, flexuous, interwoven, 2.5–5 µm in diam.

##### Tubes.

Generative hyphae hyaline, thin- to slightly thick-walled, occasionally branched, flexuous to straight, interwoven, 2–4 µm in diam. Cystidia absent; cystidioles present, clavate to fusoid, thin-walled, smooth, 16–24 × 4–5.5 µm; basidia clavate, bearing four sterigmata and a basal clamp connection, 21–26 × 5–7 μm; basidioles similar in shape to basidia, but smaller.

##### Spores.

Basidiospores broadly ellipsoid, hyaline, thin-walled, smooth, some with one or two guttules, IKI–, CB–, (3.3–)3.4–4(–4.1) × 2.5–3 μm, L = 3.69 μm, W = 2.76 μm, Q=1.34 (n = 30/1).

##### Ecology and distribution.

Growing on living tree of *Quercusmongolica*. Known from the type location only.

##### Type of rot.

White rot.

##### Specimens examined/studied.

The holotype.

#### ﻿ Hapalopilus tabuliformis

Taxon classificationFungiPolyporalesPhanerochaetaceae

Y.C. Dai, Xin Zhang, Ghobad-Nejhad & Yuan Yuan sp. nov.

508BF0A9-0B6D-558F-B08B-2CDD25E60B60

853593

Figs 4 , 5

##### Holotype.

China. Inner Mongolia Autonomous Region, Alxa County, Beisi Forest Park, on fallen branch of *Pinustabuliformis*, 18 September 2022, Dai 24540 (BJFC 039782).

**Figure 4.**
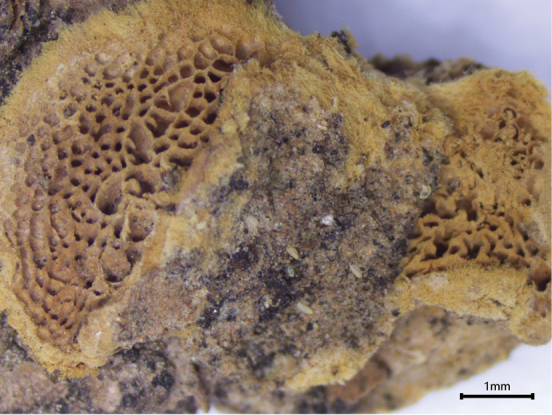
Basidiomata of *Hapalopilustabuliformis* (Dai 24540).

##### Etymology.

*Tabuliformis* (Lat.): refers to the species growing on *Pinustabuliformis*.

**Figure 5.**
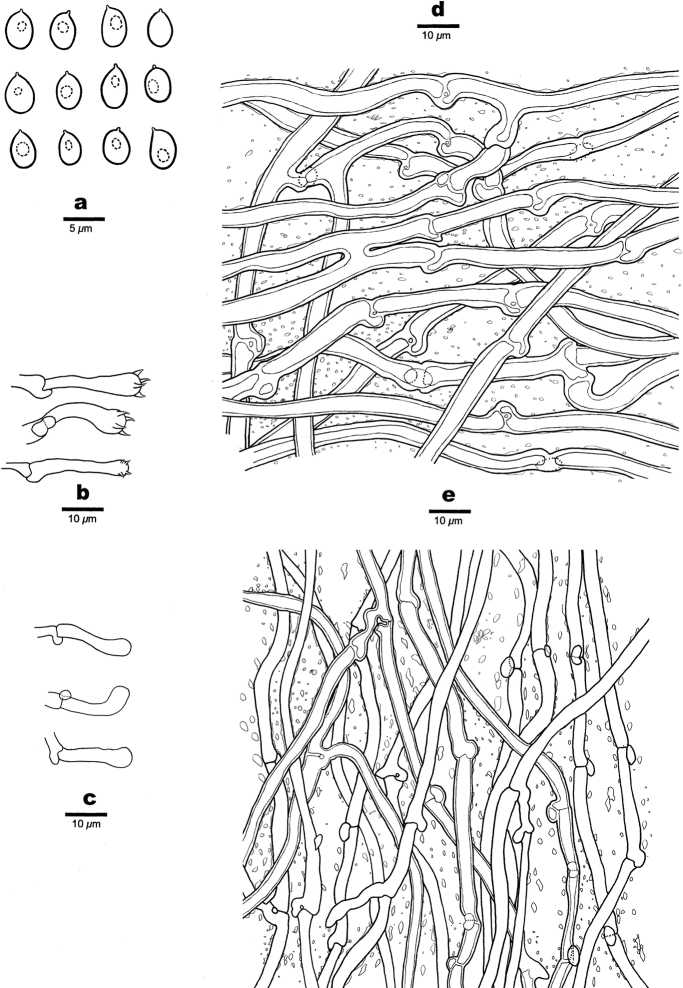
Microscopic structures of *Hapalopilustabuliformis* (Dai 24540, holotype) **a** basidiospores **b** basidia **c** basidioles, **d** hyphae from context **e** hyphae from tube trama.

##### Diagnosis.

*Hapalopilustabuliformis* is characterized by resupinate to effused-reflexed basidiomata having a pale pink to buff-yellow pileal surface and purple coloration in KOH solution, small pores 3–5 per mm, the absence of cystidioles, long and narrow basidia measuring 18–31 × 3.2–5.8 μm, broadly ellipsoid basidiospores measuring 3.2–4 × 2.6–3.2 μm, and growing on *Pinustabuliformis* in western China.

##### Fruitbody.

Basidiomata annual, resupinate to effused-reflexed, adnate, soft corky and without odor or taste when fresh, becoming brittle to hard corky upon drying. Pilei projecting up to 0.9 cm, 1.2 cm wide and 3 mm thick at base. Pileal surface pale pink to buff-yellow when fresh, becoming honey-yellow when dry. Pore surface light vinaceous gray to grayish violet when fresh, becoming buff to grayish brown when dry; margin cream to pale ochraceous, fimbriate and thinning out when resupinate, up to 1 mm wide; pores angular to irregular, 3–5 per mm; dissepiments thin, entire to lacerate. Context honey and corky when dry, up to 2 mm thick, becoming purple in KOH solution. Tube layer concolorous with pore surface, corky, up to 1 mm deep.

##### Hyphal structure.

Hyphal system monomitic; generative hyphae bearing clamp connections, richly encrusted with fine yellowish crystals (dissolved in KOH solution), IKI–, CB–; tissue becoming purple in KOH solution.

##### Context.

Generative hyphae hyaline, slightly thick- to thick-walled, occasionally branched, interwoven, 2–4.3 µm in diam.

##### Tubes.

Generative hyphae hyaline, thin- to slightly thick-walled, frequently branched, interwoven, flexuous, 3–5.9 µm in diam. Cystidia and cystidioles absent. Basidia clavate to pyriform, bearing four sterigmata and a basal clamp connection, 18–31 × 3.2–5.8 μm; basidioles similar in shape to basidia, but smaller.

##### Spores.

Basidiospores broadly ellipsoid, hyaline, thin-walled, smooth, usually with a guttule, IKI–, CB–, (3–)3.2–4(–4.2) × (2.5–)2.6–3.2(–3.4) μm, L= 3.68 μm, W = 2.76 μm, Q=1.25 (n=60/2).

##### Ecology and distribution.

Growing on fallen branches of *Pinustabuliformis*. Known from the type location only.

##### Type of rot.

White rot.

##### Additional specimen examined.

China. Inner Mongolia Autonomous Region, Alxa County, Beisi Forest Park, on fallen branch of *Pinustabuliformis*, 18 September 2022, Dai 24535 (BJFC 039777).

#### ﻿ Aurantiporus alboaurantius

Taxon classificationFungiPolyporalesMeruliaceae

(C.L. Zhao, B.K. Cui & Y.C. Dai) Y.C. Dai, Xin Zhang, Ghobad-Nejhad & Yuan Yuan comb. nov.

F66D6338-D647-5149-8545-A6C6278D0907

853599

Ceriporiopsis alboaurantia C.L. Zhao, B.K. Cui & Y.C. Dai, Phytotaxa 164: 22 (2014) (Basionym)
≡ Phlebicolorataalboaurantia (C.L. Zhao, B.K. Cui & Y.C. Dai) C.L. Zhao, J. Fungi 9 (3, no. 320): 32 (2023)

##### Description.

See Cui and Zhao (2014).

##### Ecology and distribution.

Growing on fallen trunk of *Cunninghamia*. Known from subtropical forests in southeast China.

##### Type of rot.

White rot.

##### Notes.

*Ceriporiopsispseudoplacenta* Vlasák & Ryvarden and *C.alboaurantia* were recently described from USA (Vlasák et al. 2012) and China (Cui and Zhao 2014), respectively. However, Vampola and Vlasák (2021) recombined *C.pseudoplacenta* into *Aurantiporus* following morphological analyses, and they considered *Aurantiporuspriscus* Niemelä et al. described from Europe two months later (Niemelä et al. 2012) as a taxonomic synonym of *A.pseudoplacentus* (Vlasák & Ryvarden) J. Vlasák & P. Vampola. Our updated phylogeny with enhanced taxon sampling also indicates that both species are nested within the *Aurantiporus* s. str. clade (Fig. 1), but it is not sure that they are conspecific. All these species resemble the type species of *Aurantiporus* by sharing dense agglutinated tubes, shrinking and darkening upon drying, and a monomitic hyphal system with ellipsoid, smooth basidiospores. Hence, the above combination is proposed.

*Phlebicolorata* C.L. Zhao, typified with *P.brevispora* (Nakasone) C.L. Zhao, was established to include the generic type and *A.pseudoplacentus*, *C.alboaurantia*, and *A.roseus* (C.L. Zhao & Y.C. Dai) Zmitr. (Zhao et al. 2023). However, the latter three species are nested in the *Aurantiporus* s. str. clade in our phylogeny (Fig. 1). Similar results were obtained by Liu et al. (2022).

#### ﻿ Aurantiporus mutans

Taxon classificationFungiPolyporalesMeruliaceae

(Peck) Y.C. Dai, Xin Zhang, Vlasák, Ghobad-Nejhad & Yuan Yuan comb. nov.

7752EC4C-0F24-5E0D-9F2E-86A0AE5FA818

854371

Polyporus mutans Peck, Rep. (Annual) Trustees State Mus. Nat. Hist., New York 41: 77 (1888) (Basionym)
≡ Poriamutans (Peck) Peck, Ann. Rep. Reg. N.Y. St. Mus. 43: 85 (1890)
≡ Hapalopilusmutans (Peck) Gilb. & Ryvarden, N. Amer. Polyp., Vol. 1 Abortiporus - Lindtneri (Oslo): 337 (1986)

##### Description.

See Gilbertson and Ryvarden (1986).

##### Ecology and distribution.

Growing on dead hardwoods, usually on *Castanea*. Known from eastern North America from Canada to Florida and Australia.

#### ﻿ Aurantiporus tropicus

Taxon classificationFungiPolyporalesMeruliaceae

(I. Lindblad & Ryvarden) Y.C. Dai, Xin Zhang, Vlasák, Ghobad-Nejhad & Yuan Yuan comb. nov.

7E1B4E65-440E-54B5-8EB8-300323C1673A

854372

Hapalopilus tropicus I. Lindblad & Ryvarden, Mycotaxon 71: 342 (1999) (Basionym)

##### Description.

See Lindblad and Ryvarden (1999).

##### Ecology and distribution.

Growing on dead deciduous wood. Known from tropical wet forests in Costa Rica.

##### Notes.

*Hapalopilusmutans* was first described as *Polyporusmutans* from New York, USA, and was recognized by resupinate, colorful basidiomata with a reddish coloration in KOH solution (Lowe 1966; Gilbertson and Ryvarden 1986). *Hapalopilustropicus* was originally described from the tropical forests of Costa Rica, and unlike the other *Hapalopilus* species, it is not reactive in KOH solution (Lindblad and Ryvarden 1999). However, *Hapalopilustropicus* mostly resembles *H.mutans* by having resupinate, colorful basidiomata that turn red upon bruising, dense agglutinated tubes, shrinking and darkening upon drying, and a monomitic hyphal system with ellipsoid, smooth, thin-walled basidiospores (Lowe 1966; Gilbertson and Ryvarden 1986; Lindblad and Ryvarden 1999). However, according to the present study (see discussion), the above morphological characteristics fit the definition of *Aurantiporus*. Moreover, our phylogeny (Fig. 1) confirms that *Hapalopilusmutans* and *H.tropicus* grouped together with *Aurantiporusroseus* within the *Aurantiporus* s. str. clade, which was distant from *H.rutilans* (the type of *Hapalopilus*). Thus, the above combinations are proposed.

#### ﻿ Luteoporia albocitrina

Taxon classificationFungiPolyporalesMeruliaceae

(Petch) Y.C. Dai, Xin Zhang, Vlasák, Ghobad-Nejhad & Yuan Yuan comb. nov.

213BA816-8162-5513-B48B-1E9A6EDB00A8

854431

Poria albocitrina Petch, Ann. R. bot. Gdns Peradeniya 7(4): 286 (1922) (Basionym)
≡ Hapalopilusalbocitrinus (Petch) Ryvarden, *in* Ryvarden & Johansen, Prelim. Polyp. Fl. E. Afr. (Oslo): 359 (1980)
= Luteoporiacitriniporia Z.B. Liu & Yuan Yuan, Phytotaxa 46(1): 36 (2020)

##### Description.

See Ryvarden and Johansen (1980) and Liu and Yuan (2020).

##### Ecology and distribution.

Growing on dead deciduous wood. Known from Costa Rica, Rwanda, Kenya and Sri Lanka.

##### Notes.

*Hapalopilusalbocitrinus* is a tropical species originally described as *Poriaalbocitrina* from Sri Lanka (Petch 1922), and it is characterized by resupinate, bright-colored basidiomata with a reddish coloration in KOH solution, swollen hyphae covered with crystals at the tips and cylindrical to oblong ellipsoid basidiospores (Petch 1922; Ryvarden and Johansen 1980). *Luteoporiacitriniporia* is not only morphologically similar to *H.albocitrinus*, but also have an overlapping distribution. So, we consider that *Luteoporiacitriniporia* and *H.albocitrinus* represent a single species, hence the above recombination is proposed.

##### Specimens examined.

*Aurantiporusalboaurantius*: China. Fujian Province, Wuyishan County, Longfenggu Forest Park, alt. 500 m, on fallen trunk of *Cunninghamia*, 27 August 2006, Cui 4136 (BJFC 000412, holotype); Longchuan Valley, alt. 500 m, on fallen trunk of *Cunninghamia*, 16 October 2005, Cui 2877 (BJFC 000416, paratype). *A.mutans*: USA, Pennsylvania, Wilkes-Barre, Ricketts Glen State Park, on black cherry, 11 September 2003, JV 0309/83a,b (JV, PRM); Pike County, Promised Land State Park, on *Quercus* sp., 13 September 2005, JV 0509/123 (JV, PRM). *A.pseudoplacentus*: USA, Washington, Forks, Bogachiel State Park, on trunk of *Piceasitchensis*, 6 August 2003, JV0308/68 (PRM 899297, holotype; BJFC 020510, isotype). *A.tropicus*: Costa Rica, Puntarenas Province, Santa Elena, JV1707/5-T (JV, PRM). *Luteoporiaalbocitrina*: Sri Lanka. Colombo, Dombagaskanola Forest Reserve, on rotten angiosperm wood, 27 February 2019, Dai 19507 (BJFC 031186); Avissawella, Salgala Forest, on rotten angiosperm wood, 3 March 2019, Dai 19622 (BJFC0 31299); Costa Rica, Puntarenas Province, Tarcoles, on rotten angiosperm wood, 22 April 2017, JV 1704/103 (JV).

## ﻿Discussion

Despite the controversial history of *Aurantiporus* and *Hapalopilus*, there is certainty in the placement of the new species *Aurantiporusorientalis* and *Hapalopilustabuliformis*. This placement is consistent with the type species of their corresponding genera (Fig. 1). Both species are found in the temperate forests of China and show bright-colored basidiomata with a reddish coloration in KOH solution.

Our phylogenetic analysis (Fig. 1) corroborates that *Erastia* and *Hapalopilus* are monophyletic and nested in different clades of Polyporales. *Erastia* was established to accommodate *Hapalopilus* species growing on coniferous wood (Niemelä et al. 2005), and it is nested in Irpicaceae including its type and two additional species in our phylogeny (Fig. 1.). Conversely, *Hapalopilus* is nested in Phanerochaetaceae as monophyletic clade which is in accordance with Miettinen et al. (2016), and includes five species here. In conclusion, *Erastia* and *Hapalopilus* are independent genera within Irpicaceae and Phanerochaetaceae, respectively.

*Hapalopilustabuliformis* is an independent lineage within *Hapalopilus*, as indicated by the phylogenetic analysis of the combined ITS1-5.8S-ITS2-nLSU dataset (Fig. 1). Morphologically, *H.eupatorii* (P. Karst.) Spirin & Miettinen resembles *H.tabuliformis* by having resupinate to effused-reflexed basidiomata, fimbriate margin and similar sized ellipsoid basidiospores, but differs from *H.tabuliformis* by shorter basidia (14–18 µm vs. 18–31 μm) and longer basidiospores (3.4–4.5 µm vs. 3.2–4 µm; Miettinen et al. 2016; Zíbarová et al. 2021). *Hapalopilusrutilans* and *H.ribicola* (P. Karst.) Spirin & Miettinen differ from *H.tabuliformis* by basidiospore sizes (4–5 × 2.3–3 µm in *H.ribicola* and 3.2–5.1 × 2–2.7 µm in *H.rutilans* vs. 3.2–4 × 2.6–3.2 μm in *H.tabuliformis*) and shorter basidia (16.5–20.5 × 4.5–6 µm in *H.ribicola* and 18–22 × 5–6.5 µm in *H.rutilans* vs. 18–31 × 3.2–5.8 μm in *H.tabuliformis*; Miettinen et al. 2016; Ryvarden and Melo 2017). Moreover, the former two species have a wide distribution in Europe and commonly grow on deciduous trees (Miettinen et al. 2016), while *H.tabuliformis* is known from China and grow on *Pinus*. *Hapalopiluspercoctus* differs from *H.tabuliformis* by having pileate basidiomata, longer basidiospores (3.8–4.6 µm vs. 3.2–4 µm), and is known to grow on dicots in the Southern Hemisphere (Miettinen et al. 2016).

*Aurantiporus* is found to be highly polyphyletic in the family Meruliaceae, as shown in our phylogenetic analysis as well as in previous studies (Floudas and Hibbett 2015; Chen et al. 2021; Liu et al. 2022). The type species of *Aurantiporus* was erected by Murrill (1905) as *Polyporuspilotae* Schwein. (Schweinitz 1832) described from North America, and was later considered by himself as a synonym of *Polyporuscroceus* (Pers.) Fr. (=*Boletuscroceus* Pers.; Persoon 1796; Fries 1815). Nevertheless, it is noteworthy that the sequences sampled from North America and Europe, and named as *Hapalopiluscroceus* in GenBank, represent a species complex (Fig. 1). We do not doubt that *Aurantiporuscroceus* or *A.pilotae* (Schwein.) Murrill belong to *Aurantiporus* rather than *Hapalopilus*. In addition, similar uncertainty exists within *Aurantiporuspriscus*, which was synonymized as *A.pseudoplacentus* by Vampola and Vlasák (2021). Our phylogenetic analysis illustrated that they make form two closely related separate subclades (Fig. 1). However, *Aurantiporuspriscus* in our phylogeny is represented by specimens from China and Russia Far East, with no sequences available from the type locality (Poland). So, further study is needed, especially the sequences from type locality are very important to confirm the species complex.

The new species *Aurantiporusorientalis* nested in the *Aurantiporus* s. str. clade (Fig. 1) and grouped together with the generic type (*A.pilotae* from North America, Fig. 1). Morphologically, these species share pileate, bright orange-red colored basidiomata and grow on *Quercus*. However, the generic type (*A.pilotae* from North America) differs from *A.orientalis* by the absence of cystidioles (Murrill 1905; Koszka and Papp 2020). *Aurantiporuspseudoplacentus*, *A.mutans*, and *A.tropicus* are similar to *A.orientalis* in having orangish basidiomata and similar shape and size of basidiospores, but *A.pseudoplacentus*, *A.mutans*, and *A.tropicus* differ from *A.orientalis* by the absence of cystidioles (Lowe 1966; Gilbertson and Ryvarden 1986; Lindblad and Ryvarden 1999; Vlasák et al. 2012). In addition, *A.alboaurantius*, *A.roseus*, and *A.orientalis* share a monomitic hyphal system with ellipsoid, thin-walled basidiospores and the presence of cystidioles (Cui and Zhao 2014; Zhao et al. 2015). However, *A.alboaurantius* and *A.roseus* differ from *A.orientalis* in having resupinate basidiomata and bigger basidiospores (4–5 × 3–3.3 µm in *A.alboaurantius* and 4–5.2 × 3.3–3.8 µm in *A.roseus* vs. 3.4–4 × 2.5–3 µm in *A.orientalis*; Cui and Zhao 2014; Zhao et al. 2015).

From a morphological perspective, *Hapalopilus* is characterized by pileate to resupinate, colorful, and soft to cottony corky basidiomata when fresh, brittle when dry, a monomitic hyphal system with generative hyphae bearing clamp connections, and covered with granular, golden yellow pigment that dissolves in KOH solution (Miettinen et al. 2016). *Aurantiporus* s. str. differs from *Hapalopilus* by having larger, watery and fleshy basidiomata when fresh, slower drying process, often shrinking significantly in size, denser agglutinated tubes, darker and hard when dried, generative hyphae somewhat colored, and usually covered with oily matter not dissolving in KOH solution (Jahn 1974; Zmitrovich et al. 2006; Wu et al. 2010; Niemelä et al. 2012; Vlasák et al. 2012). Moreover, the traditional concept of *Aurantiporus* has caused difficulty in defining certain species, such as *A.pulcherrimus* (Rodway) P.K. Buchanan & Hood, *A.fissilis* (Berk. & M.A. Curtis) H. Jahn, and *A.alborubescens* (Romell) H. Jahn, which were also combined into the genus *Tyromyces* P. Karst. due to morphological similarities (Reid 1967; Yao et al. 1999; Buchanan and Ryvarden 2000; Ryvarden and Melo 2017). These species seem to be phylogenetically distant from the type species of *Aurantiporus*, *Hapalopilus*, and *Tyromyces* (Binder et al. 2013; Floudas and Hibbett 2015; Liu et al. 2023). Recently, the genus *Odoria* V. Papp & Dima was established to accommodate *A.alborubescens* (Papp and Dima 2017), and *Pappia* Zmitr. was established for *A.fissilis* (Zmitrovich 2018), while the phylogenetic affiliations of other *Aurantiporus* species remained unclear. Our current phylogeny strongly supports the treatment of *A.fissilis* in the monophyletic genus *Pappia*. Additionally, the distinctive characteristics of whitish pileate basidiomata, shrinking significantly in size when dry, a pleasant and sweet smell, and the presence of chlamydospores, differentiate *Pappiafissilis* as a unique entity in both *Aurantiporus* and *Tyromyces* (Ryvarden and Melo 2017; Chen et al. 2021).

The genus *Aurantiopileus* D.L. Lindner & T.J. Baroni, typified as *A.mayaensis* Ginns, was erected by Ginns et al. (2010), and unlike *Aurantiporus*, it has cystidia. Confusingly, *Aurantiopileusmayaensis* was somehow combined into *Aurantiporus* by Zmitrovich (2018). In our phylogenetic analysis (Fig. 1), the two genera *sensu typi* stand apart from each other and are clustered in different clades. Nevertheless, *A.mayaensis* is nested with *Aurantiporusalbidus* Rajchenb. & Cwielong described from Argentina, and both species are characterized by large, watery, and fleshy basidiomata when fresh, shrink and hard when dried which fit well with the concept of *Aurantiporus* in morphology (Rajchenberg 1995; Ginns et al. 2010). Regarding the available GenBank sequences attributed to *Aurantiporusalbidus*, two Australian specimens (Cui 16664 and Cui 16665) and two Argentinean specimens (CIEFAP-117 and F32) formed two lineages in our phylogeny (Fig. 1). It seems that the Australian samples represent another taxon rather than *Aurantiporusalbidus*. Notably, *Aurantiporusalbidus* stands out microscopically from other *Aurantiporus* species in its strongly agglutinated generative hyphae covered with hyaline, resinous material and thick-walled, amyloid basidiospores, and may be a distinct species as suggested by Rajchenberg in (1995). However, it is certain that taxa of *Aurantiopileus* and *Aurantiporus* s. str. nested in two clades in Meruliaceae.

## Supplementary Material

XML Treatment for Aurantiporus orientalis

XML Treatment for Hapalopilus tabuliformis

XML Treatment for Aurantiporus alboaurantius

XML Treatment for Aurantiporus mutans

XML Treatment for Aurantiporus tropicus

XML Treatment for Luteoporia albocitrina

## ﻿Additional information

### Author ORCIDs

Xin Zhang https://orcid.org/0009-0005-8363-7852

Hong-Min Zhou https://orcid.org/0000-0002-0724-5815

Masoomeh Ghobad-Nejhad https://orcid.org/0000-0002-7807-4187

Hong-Gao Liu https://orcid.org/0000-0002-9508-3245

Josef Vlasák https://orcid.org/0000-0002-5363-4459

Yu-Cheng Dai https://orcid.org/0000-0002-6523-0320

Yuan Yuan https://orcid.org/0000-0001-6674-9848

### Data availability

All of the data that support the findings of this study are available in the main text.
